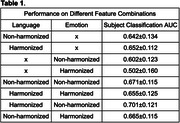# Subject Harmonization of Multi‐Modal Digital Markers: Improved Detection of Mild Cognitive Impairment Using Language and Facial Expression

**DOI:** 10.1002/alz.094211

**Published:** 2025-01-09

**Authors:** Bao Hoang, Yijiang Pang, Hiroko H Dodge, Jiayu Zhou

**Affiliations:** ^1^ Michigan State University, East Lansing, MI USA; ^2^ Massachusetts General Hospital, Harvard Medical School, Boston, MA USA

## Abstract

**Background:**

Mild Cognitive Impairment (MCI) is the prodromal stage of dementia, including Alzheimer’s Disease (AD). Early identification and accurate assessment of MCI are critical for clinical trial enrichment as well as the early intervention of AD. Digital makers offered a unique opportunity for ecologically valid and affordable early detection approaches. Language markers extracted from verbal communications have shown diagnostic efficacy in detecting early MCI. Recent studies have shown that in addition to semantic and syntactic information in dialogues, emotions in communication can also be helpful in early MCI detection. A joint analysis of language markers and emotion indicative of facial expression is thus of great interest. Features from emotion could have additional predictive benefits to language markers. One general challenge of digital biomarkers is that feature distributions are very distinct. We hereby conduct a multi‐modal analysis of language and facial expression, combined with different harmonization.

**Method:**

We used 3,501 conversations from 69 participants from the Internet‐Based Conversational Engagement Clinical Trial (I‐CONECT) (NCT02871921) and extracted language and emotion variables. For language variables, we used 99‐dimensional features from four types: Linguistic Inquiry and Word Count (LIWC) (64), Syntactic Complexity (23), Lexical Diversity (10), and Response Length (2). For emotion features, we extracted a 7‐dimensional emotion feature (angry, disgust, fear, happy, sad, surprise, neutral) from a facial frame using the DeepFace library. For analysis, we used the subject harmonization approach to remove information that is predictive of subjects. The harmonized features are then used to build a neural network for MCI detection.

**Result:**

We evaluated Area‐under‐Curve (AUC) on subject classification. Our results show that applying harmonization will increase detection performance when only using language markers. If we harmonize both language and emotion features, the performance decreases. However, if we do harmonization on language and without harmonization on emotion, then the performance of subject classification increases to 0.701 AUC.

**Conclusion:**

Our study has shown the additional benefits of emotion variables to augment language variables in the early detection of MCI. Multimodality analyses need careful selection of harmonization: harmonization strategy should be chosen for individual modality rather than the concatenated feature variables.